# Hydration and Expansion Characteristics of MgO Expansive Agent in Mass Concrete

**DOI:** 10.3390/ma15228028

**Published:** 2022-11-14

**Authors:** Feifei Jiang, Zhongyang Mao, Lanqing Yu

**Affiliations:** 1 Suzhou Institute of Technology, Jiangsu University of Science and Technology, Suzhou 215600, China; 2 State Key Laboratory of Materials-Oriented Chemical Engineering, College of Materials Science and Engineering, Nanjing Tech University, Nanjing 211800, China

**Keywords:** concrete shrinkage, MgO expansive agent, reinforced concrete wall, volumetric deformation

## Abstract

Based on the underground reinforced concrete wall of subway stations (Hangzhou, China), this paper studied the influence of a MgO expansive agent (MEA) on deformation and mechanical properties of a reinforced concrete wall. The results show that the effect of the MEA with different activities to compensate for the shrinkage of reinforced concrete walls is different. For MEA-R (60 s), because the activity is too high, its hydration rate is too fast, and many expansions occur at the plastic state of the concrete, which cannot effectively compensate for the shrinkage of concrete. For MEA-S (220 s), due to its low activity, the early hydration rate is so slow that it cannot compensate for the shrinkage, but it compensates well at the later stage due to the continuous hydration expansion of MEA. For MEA-M (140 s), the shrinkage of concrete is well compensated for the shrinkage at the early, middle and late stages due to its moderate activity. After using MEA to partially replace fly ash and mineral powder, the compressive strength of concrete was lower at the early stage (0–28 days). However, in the later stage, the porosity of concrete decreased rapidly, and the compressive strength of concrete would also be significantly improved. Therefore, choosing a suitably active MEA can compensate for the shrinkage of mass concrete without reducing its strength.

## 1. Introduction

Cracks caused by non-load deformation in concrete affect the durability of buildings and seriously endanger the safety of buildings [1]. In recent years, the acceleration of urbanization and the rapid development of infrastructure construction in China have put forward higher requirements on the long-term durability of concrete. On the one hand, there are many kinds of raw materials used in concrete and their properties fluctuate greatly, and more mineral admixtures are added. Coupled with the lack of reasonable maintenance measures, the shrinkage deformation of concrete has been seriously intensified. On the other hand, practitioners do not fully understand the characteristics of concrete materials, and engineers in design and construction also lack relevant guidance on effective crack control technology, which further affects the long-term durability of the current high-performance concrete in China [2,3].

In recent years, many engineers and researchers have studied the structural cracking caused by the shrinkage of cement-based materials. There are five traditional ways to reduce shrinkage or cracking of cement-based materials. The first method is to replace part of cement to reduce the hydration heat of cement-based materials by mixing with mineral addition (milled fine fly ash or slag powder) [4]. The second method is to increase the elastic modulus of concrete by adding steel fibers [5]. The third method is to improve the tensile strength and toughness of concrete by adding polypropylene fibers [6]. The fourth method is to reduce the capillary pressure of concrete during water loss and reduce its drying shrinkage by adding shrinkage-reducing admixtures [7]. The fifth method is to compensate for the shrinkage of concrete with the addition of expansive agent. Among all the above-mentioned methods, adding an expansive agent is considered to be one of the most effective methods to prevent concrete from cracking [8,9]. The types of expansive agents are different, and the quality of different batches of the same expansive agent also varies greatly, which seriously affects the use effect of the expansive agent. Traditional expansive agents mainly include sulphate-aluminate, CaO-based and MgO-based expansive agents (MEA) [10,11,12]. Even though sulphate-aluminate and CaO-based agents have got a lot of application in the field of construction engineering for many years, these two kinds of expansive agents still have many defects which cannot be overcome by themselves. For example, sulphate-aluminate and CaO-based expansive agents mainly produce expansion within 3–14 days after casting and produce almost no expansion after 28 days. However, the shrinkage of concrete is accompanied by the whole process of cement hydration, which may last for half a year or as long as several years. At the later stage, these two kinds of expansive agents (sulphate-aluminate and CaO-based expansive agents) cannot produce enough expansion to compensate for the shrinkage of the concrete. Their expansion properties are poorly adjustable, and the expansion performance cannot be adjusted and designed according to the shrinkage characteristics of the target concrete. Moreover, the hydration product of sulphate-aluminate expansive agent in reaction with water is ettringite (AFt). AFt has 32 crystal waters, which indicates that adequate water is needed for the reaction of the sulphate-aluminate expansive agent. When the high-strength concrete is in a dry condition with no humidity exchange and low water–binder ratio, the expansion performance of the sulphate-aluminate expansive agent is restricted by insufficient water. What is more, the physical and chemical properties of AFt are not stable. It is easy to decompose at about 75 °C, and it is easy to dissolve under pressure, which will make the sulphate-aluminate expansive agent lose its expansion effect [13,14,15,16,17]. Compared with other expansive agents, MEAs requires less water when reacting with water. Additionally, the hydration product of MgO is Mg(OH)_2_, which is more stable. In addition, MEAs with different expansion properties can be produced through artificial control calcination temperature to meet the needs of different projects [18,19]. For these reasons, MEAs have been gradually developed into one of the most commonly used expansive agents in China.

Based on the results of previous researchers, after MgO hydrates to form Mg(OH)_2_, the solid phase volume increases by 118%, so the expansion force generated by MgO in cement-based materials can compensate concrete shrinkage [20]. The low-activity MEA prepared by calcination at high temperature (1000–1200 °C) has delayed expansion characteristics and has been widely used in mass concrete such as dams and hydropower stations to compensate for several months of temperature drop shrinkage in concrete. However, such structures are plain concrete without steel reinforcement, and there are few studies on the performance and application effect of reinforced concrete. For civil buildings, MEAs have not been widely used in concrete crack resistance and water penetration prevention The main reason is that people’s understanding of MEAs is still limited to the characteristics of delayed expansion, resulting in many successful cases of application of MEA in dam concrete, but less in other projects. At the same time, there have been doubts about the safety of MEAs. The activity of MgO in cement after calcination at high temperature (1300–1450 °C) is extremely low, so that its hydration rate is very slow, and it is easy to produce harmful expansion in the later stage, so the MgO content in cement clinker cannot exceed 5% in China’s specifications [21]. This seriously restricts the promotion and application of MEAs. Currently, there are few cases of applying MEAs to civil construction projects. Due to insufficient data, it is difficult to promote the application of MEAs in these fields. The temperature drop shrinkage of concrete in civil buildings occurs earlier and the temperature drop speed is fast. Therefore, the requirement for an expansive agent is that it can produce sufficient expansion at the early stage, and relatively mild expansion at the later stage. However, the low-activity MEA used in dams in the past cannot effectively meet these needs.

In fact, the reaction rate and expansion performance of MEAs are closely related to its activity. By changing its calcination temperature and holding time, MEAs with different expansion properties can be produced. According to the preliminary study of our group, MEAs with high activity produced at a low temperature have a higher expansion rate at the early stage but a lower expansion rate at the later stage [20]. On the other hand, MEAs with low activity produced at a high temperature have a lower expansion rate at the early stage but a higher expansion rate at the later stage. Therefore, an MEA calcined at a lower temperature (80–1000 °C), namely, lightly burnt MEA (LBMEA), has a larger early expansion and can theoretically compensate for the shrinkage of concrete during early temperature drop.

In this study, the reinforced concrete wall of the Hangzhou subway station is used as the research object. The volume deformation properties and the mechanical properties of MEA concrete under the conditions of conservation of natural conditions and rebar constraints were studied, and the hydration and deformation laws of MEAs with different activities were revealed under the condition of rebar constraints. The temperature change process and volume deformation of the reinforced concrete wall in outdoor environments were recorded by the vibration strain gauges, the humidity meter was used to measure the internal humidity of the concrete, and the degree of hydration of the MEA in the internal concrete wall was measured by thermal analysis.

## 2. Materials and Experiments

### 2.1. Materials

Portland cement, fly ash, coarse aggregates, fine aggregates, MEA and polycarboxylate-based high-range water reducer were used as the experimental objects. Details about each material are enumerated below.

#### 2.1.1. Cement

**Figure 1:**
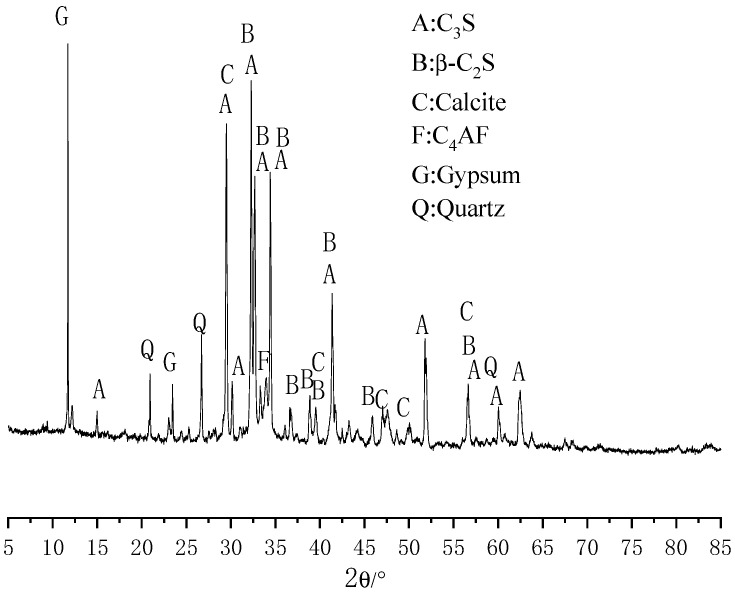
X-ray diffraction pattern of the cement.

Cement was P.O. 42.5 cement produced by Conch Group Co., Ltd. (Anhui, China). Its specific surface area is 353 m^2^/kg. Table 1 shows the detailed chemical composition of the cement, and the main chemical components were CaO, SiO_2_, Fe_2_O_3_, Al_2_O_3_ and so on. The ARL XTRA-type Cu-target X-ray diffraction instrument produced by the United States was used to analyze the material composition of the cement, with a tube voltage of 45 kV, a tube current of 35 mA, a scanning range of 5 to 85 °C, a scanning rate of 10 degrees/min and a step length of 0.02 degrees. The test results are shown in Figure 1.

#### 2.1.2. Fly Ash

Fly ash was produced by Huaneng Renewable Resources Utilization Co., Ltd. (Huai’an, China). Table 2 shows the detailed chemical composition of the fly ash, and its specific surface area is 329 m^2^/kg.

#### 2.1.3. Mineral Powder

Mineral powder was produced by Jiangsu Huailong Building Materials Co., Ltd. (Nanjing, China). Its specific surface area is 406 m^2^/kg, and its chemical composition is shown in Table 3.

#### 2.1.4. MEA

**Figure 2:**
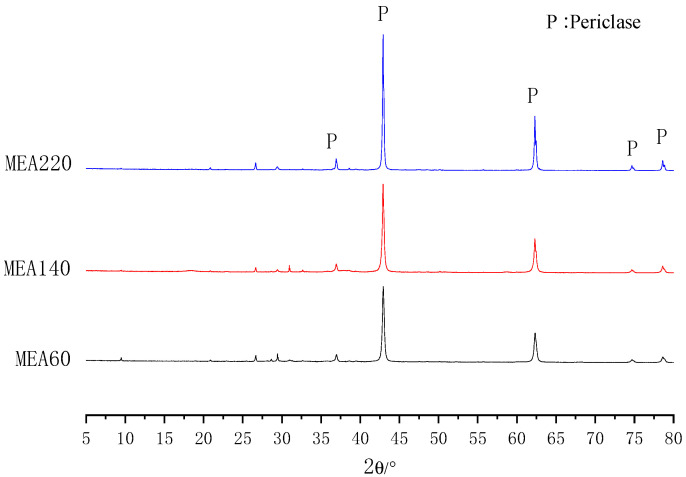
X-ray diffraction patterns of the MEAs.

**Figure 3:**
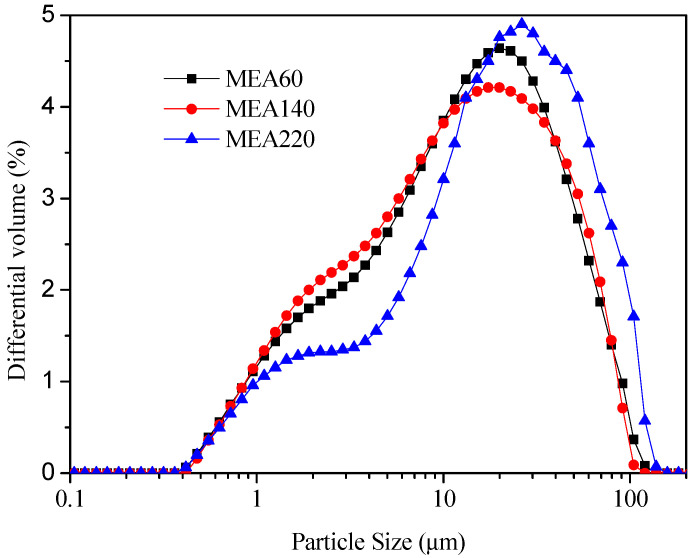
Particle size distribution of the MEAs.

In this study, three kinds of MEAs with different activities produced by Wuhan Sanyuan Special Building Materials Co., Ltd. (Wuhan, China) were used. By using a citric acid testing method, the activity values were 60 s, 140 s and 220 s, respectively, which were denoted as MEA-R, MEA-M and MEA-S. Their chemical composition is shown in Table 4, and their mineral composition is shown in Figure 2. The main component of the MEA of the three models was MgO, and the content was above 83%. There was also a small amount of CaO and SiO_2_ in the MEA. The mineral phase was mainly periclase. The results of particle size distribution are shown in Figure 3. The MEAs with three different activities had similar particle sizes, with average particle sizes of 14.073 μm, 13.080 μm and 16.450 μm, respectively. The mean particle size of the MEA with low activity was larger.

#### 2.1.5. Mix Proportions

The mix proportions of concrete are listed in Table 5. The content of the MgO expansive agent was 8% of the total amount of cementitious materials, by weight. The proportions of the cement paste (used to measure the pore structure and hydration degree) inside the wall hole is shown in Table 6.

### 2.2. Experimental Works

#### 2.2.1. Strain Gauge

Type-10 vibrating wire strain gauges made by Nanjing Gelan Industrial Co., Ltd. (Nanjing, China) were used. The gauge distance of strain gauge was 100 mm, and the vibrating modulus of the strain gauge, F, was closely related to its length, varying with the length change of the transducer brought about by the deformation of concrete [22]. The total strain of concrete was calculated by Equation (1), and the self-volumetric deformation of concrete was calculated by Equation (2).

*ε_total_* = *k* × Δ*F* + *b* × Δ*T* (1)

*ε_sv_* = *k* × Δ*F* + (*b* − *a*) × Δ*T* (2)

where *ε_total_* is the total strain of concrete; *ε_sv_* is the self-volumetric deformation of concrete; *k* is the measurement sensitivity of strain gauge; Δ*F* is the change in the measured value in real time relative to the base value of the vibrating modulus; *b* is the temperature correction factor of strain gauge; *a* is the thermal expansion coefficient of concrete, which was tested via increasing the temperature of concrete from 25 °C to 55 °C and the average value 7.9 × 10^−6^/°C was taken; Δ*T* is the change in the temperature.

#### 2.2.2. Pouring of Reinforced Concrete Wall

**Figure 4:**
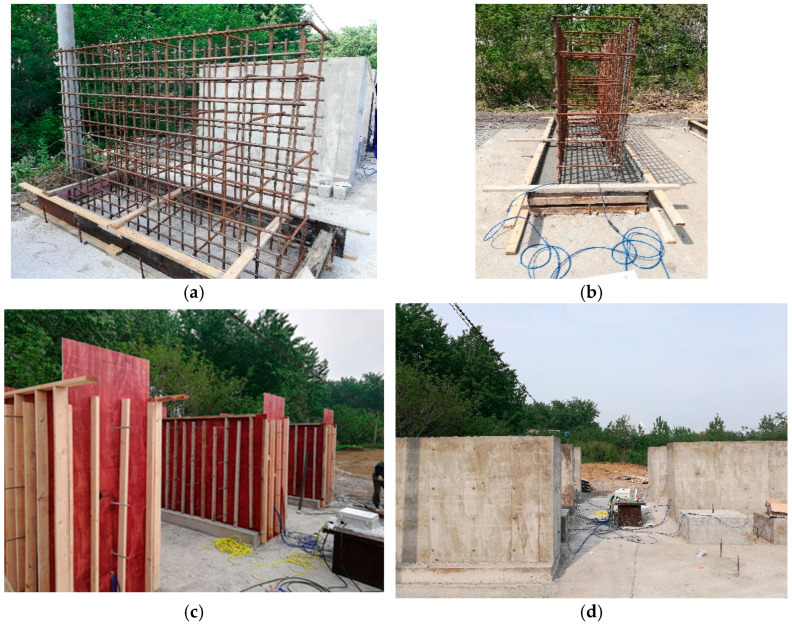
Pouring of reinforced concrete walls. (**a**) Layout of reinforcement, (**b**) Casting of foundation, (**c**) Erection of formwork, (**d**) Maintain in the outdoor environment.

**Figure 5:**
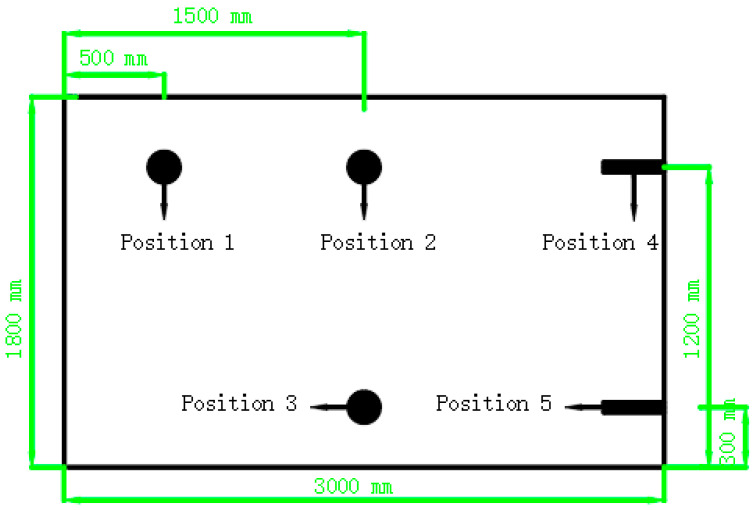
Distribution of measuring sensors.

The wall was constructed in the spring, and the atmospheric temperature at that time was 25 °C. The size of the reinforced concrete wall was 3 m × 0.8 m × 1.85 m. The thickness of the protection layer of reinforcement was 50 mm, and the spacing between horizontal reinforcement and vertical reinforcement was 150 mm. The layout of reinforcement is shown in Figure 4a. In order to simulate the constraint of foundation or baseplate on the wall in the actual construction works, foundations were prepared for all the walls in this experiment, and the size of the foundation was 0.2 m × 1.2 m × 3 m (Figure 4b). Three strain gauges were arranged in different positions inside the wall, respectively, in positions 1, 2 and 3. All strain gages are placed horizontally along the length of the wall at a distance of 50 mm from the outer surface (thickness of the rebar protective layer). The specific layout of strain gauges is shown in Figure 5. No. 1 strain gauge was used to measure the temperature and deformation at the middle height edge of the wall, No. 2 strain gauge was used to measure the temperature and deformation of the center of the middle height of the wall, and No. 3 strain gauge was used to measure the temperature and deformation of the center of the bottom height of the wall.

In order to test the hydration degree of the MEA in reinforced concrete wall, cement paste samples with the same mixing ratio were poured. When pouring concrete, a cuboid hole of 50 mm × 50 mm × 750 mm is reserved for placing the cement paste specimen. The hole for the embedded paste specimen was set at position 4. A 20 mm × 20 mm × 750 mm hole was set at position 5 for placing a humidity sensor to measure the internal humidity of the concrete wall. The HX94CNPT humidity sensors was manufactured by Omega Engineering (Norwalk, CT, USA).

#### 2.2.3. Degree of MgO Hydration in Slurry

The mass of the tested sample was reduced owing to the dehydration and decomposition of the cement-based material. According to the mass loss within the temperature range where the dehydration peak of a substance is located, the content of decomposed hydration products can be calculated. The dehydration peak of Mg(OH)_2_ in this experiment was 310~400 °C, and Equation (3) was used to calculate the content of Mg(OH)_2_ in the cement paste [23]. Thermogravimetric analysis was tested by a thermal analyzer (STA449 F3 Jupiter, Netzsch Co., Ltd. (Selb, Bavaria, Germany) under an atmosphere of nitrogen.

(3) $${Mass}_{Mg{\left(OH\right)}_{2}}=3.22\times {Mass}_{loss}\left(310\;{}^{\circ}C-400\;{}^{\circ}C\right)$$

where $Mass_{Mg{\left(OH\right)}_{2}}$ is the quantity of Mg(OH)_2_; *Mass_loss_* (310 °C–400 °C) is the mass loss at the temperature range of 320 °C–400 °C caused by the decomposition of Mg(OH)_2._

#### 2.2.4. Compressive Strength of Pumped Concrete

In the beginning, three different strength test methods were compared. The first is to directly test the strength of concrete walls with a rebound meter, which is often used on buildings in China, but its accuracy is not high. The second is to drill the specimen from the concrete wall by drilling holes, but this will destroy the integrity of the wall, which will affect the subsequent test of the deformation of the concrete wall. Finally, the method of maintaining the cube test block in the outdoor environment was adopted, which was highly accurate and did not destroy the integrity of the wall.

At the same time as pouring the concrete wall, the pumping of concrete was used to form 150 mm × 150 mm × 150 mm cube concrete blocks. The blocks were placed in the standard treatment room for one day, and then the blocks were placed near the concrete wall, so that they were in the outdoor environment. At the age of the corresponding age, the blocks were taken out to test the compressive strength. By analyzing the compressive strength of concrete with different mix proportions, the influences of the MEA activity and hydration on concrete were studied.

#### 2.2.5. Pore Distribution

At the specified age, the cement paste buried in the wall was removed, the surface part of cement paste was knocked out, and the internal part was taken to make a small test block of 2 mm^3^. For each concrete wall, two blocks were made for testing. Subsequently, the test blocks were soaked in absolute ethanol for 24 h to stop the hydration of the cement and MEA. Next, the test blocks were placed in a vacuum drying oven at 50 °C for 12 h. Finally, Mercury Intrusion Porosimetry (MIP) was used to test the pore distribution to study the effect of different active MEAs on the pore structure.

## 3. Results and Discussion

### 3.1. Temperature Change Process of the Reinforced Concrete Wall

**Figure 6:**
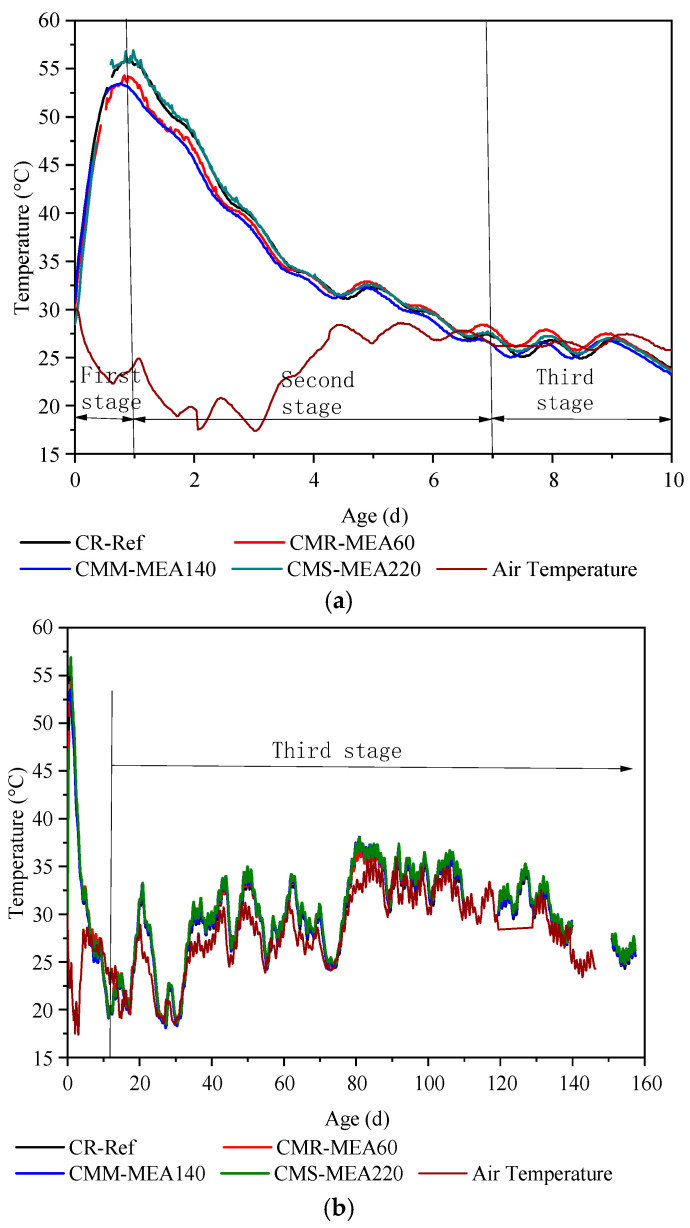
Temperature change process of reinforced concrete wall: (**a**) Temperature change from 0 to 10 days, (**b**) Temperature change from 0 to 160 days.

The temperature change process of the reinforced concrete wall could be divided into three stages, namely, the rapid temperature rise stage, rapid temperature drop stage and change stage with ambient temperature. The concrete temperature (in the depth of 5 cm) measured by three strain gauges was basically the same. In this paper, the temperature measured by No. 2 strain gauge was taken as an example to introduce the temperature variation. The detailed temperature variation is shown in Figure 6.

The first stage was the stage of intense heating. During this period of time, the hydration reaction of active components such as cement, mineral powder and MEA was intense, and the temperature of reinforced concrete wall increased sharply. This stage lasted about one day, with the highest temperature reaching 56 °C. During this period, the concrete is in the state of thermal expansion, and the stress in the concrete is the compressive stress generated by the restraint of the reinforcement and the foundation, which will not cause the concrete to crack. Comparing the data of the reinforced concrete wall in this paper with the temperature of the dam in other publications, it is found that its temperature change is very different from that of the dam [24]. For dams, the heat generated by hydration is large due to the large amount of cement. Moreover, the large width of the dam makes it difficult to dissipate the hydration heat, which makes the temperature rise of the dam continue for 1800 h. After 1800 h, the temperature gradually decreases. So, the activity of MEA in the dam needs to be low to be able to compensate for the later shrinkage. MEA140 in this paper, in contrast, can produce large expansion at an early stage.

The second stage was the rapid cooling stage. During this period of time, the rate of hydration and heat release of cement slowed down significantly. Owing to the large temperature difference between the inside and the outside, the heat from the inner core slowly diffused into the air, thus gradually cooling down. This stage lasted about 7 days, and the internal temperature of the concrete wall was significantly higher than the outside air temperature, resulting in continuous heat dissipation from the wall. In this process, the CR-Ref concrete was in a state of shrinkage due to the temperature drop, and the stress in the concrete was tensile stress owing to the constraint of the steel bar and foundation. In the process, the concrete wall would crack if the tensile stress exceeded the limit of the tensile strength.

The third stage was accompanied by the change of atmospheric temperature. During this period, the temperature of the reinforced concrete wall was basically the same as the atmospheric temperature, which changed with the atmospheric temperature.

### 3.2. Volumetric Deformation of Reinforced Concrete Wall

**Figure 7:**
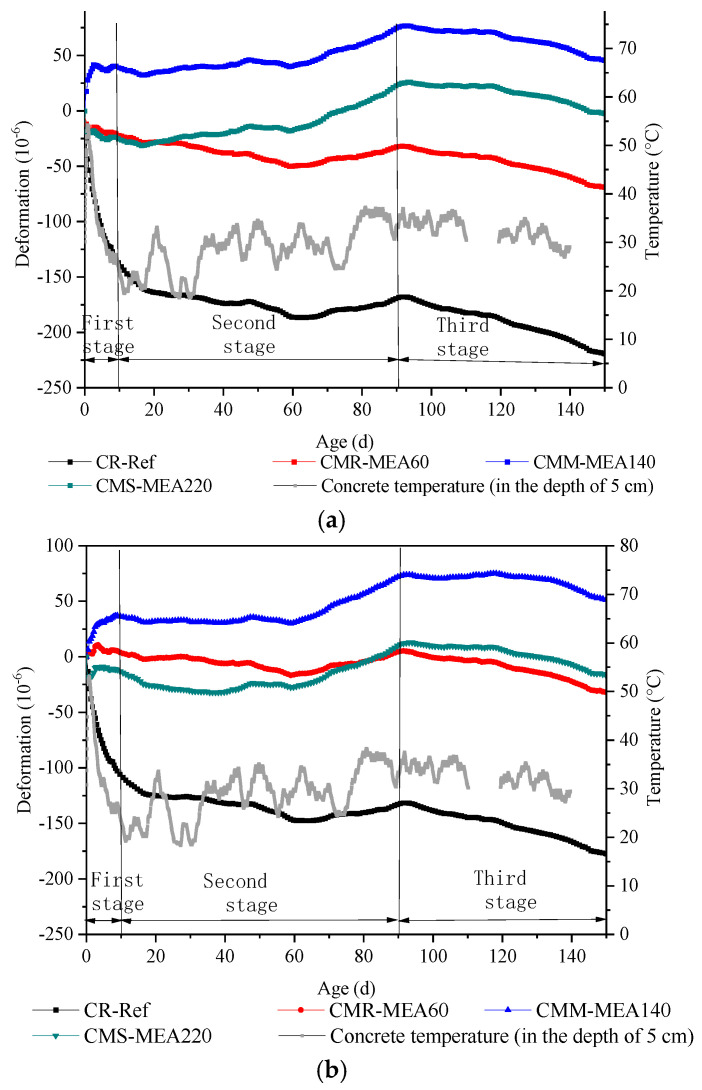

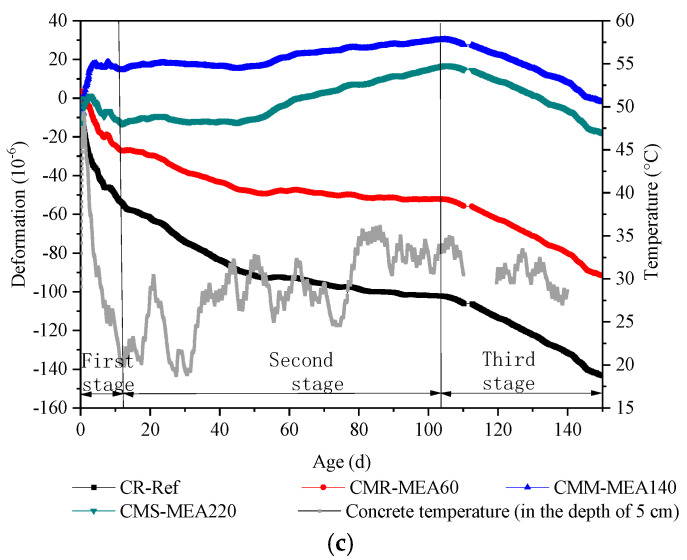
Volume deformation of reinforced concrete wall at different positions after deducting the influence of temperature: (**a**) Position 1, (**b**) Position 2, (**c**) Position 3.

Figure 7 and Table 7 show the volumetric deformation curve of concrete walls at different positions after deducting the influence of temperature. The deformation was the comprehensive result of the concrete self-contracting and expansion of the MEA. According to the measured deformation data, the whole deformation process could be divided into three stages. The first stage was a stage of drastic volume change. At this stage, the shrinkage of the concrete dominated, lasting about 10 days. The second stage, the moderate expansion stage, lasted about 90 days. The third stage was the anaphase stage of expansion.

In the first stage, intense contraction appeared in CR, producing microstrains of −138 με, −106 με, and −50 με, respectively, at three locations. This was owing to the early high temperature accelerated the hydration rate of cement, leading to greater self-shrinkage of the concrete. After the first stage, the contraction rate of CR slowed down, and the cause of CR contraction changed from self-shrinkage to dry shrinkage, finally reaching a maximum of −235 με in 150 days. On the other hand, the concrete shrinkage at three different positions was not exactly the same, and the shrinkage at position 3 was significantly less than that of the other two. This was mainly owing to the fact that position 3 was closer to the foundation, which constrained the shrinkage and deformation of the concrete, resulting in a relatively smaller shrinkage.

For concrete mixed with the MEA, it showed a completely different deformation. The CMM expanded significantly during the first phase. For example, at position 1 and position 2, the deformation was 39 με and 36 με, respectively. At position 3, owing to the foundation constrained the expansion of MEA, its expansion was reduced relative to the other two positions, and the 10 days expansion was 19 με. On the other hand, the expansion produced by CMR and CMS at this stage cannot completely compensate for the shrinkage of concrete, and the shrinkage was −7 με~−25 με.

In the second stage, the expansion of the MEA was greater than the shrinkage of concrete, resulting in micro-expansion of the reinforced concrete walls. During this period, for CMM, the expansion gradually increased, reaching a maximum value of 75 με at 90 days. At the same time, the expansion of position 3 was also smaller than that of the other two positions owing to the constraint of the foundation.

Comparing the two stages, the following conclusions can be drawn. For CMR, because the activity is too high, its hydration rate is too fast, and many expansions occur at the plastic state of the concrete, which cannot effectively compensate for the shrinkage of concrete. For CMS, due to its low activity, the early hydration rate is so slow that it cannot compensate for the shrinkage, but it compensates well at the later stage due to the continuous hydration expansion of the MEA. For CMM, the shrinkage of concrete is well compensated for the shrinkage at all the stages due to its moderate activity.

In the third stage, due to the large consumption of the MEA in the early stage, there are few remaining MEAs that can continue to produce expansion. At this stage, contraction and expansion were basically equal, and concrete expansion gradually decreased. The deformation of CMR tended to be flat and even shrink. The later deformation of CMM and CMS was still expansion deformation, which indicated that the hydration expansion of MEA-M and MEA-S could compensate for the self-shrinkage caused by hydration and the shrinkage caused by drying.

**Figure 8:**
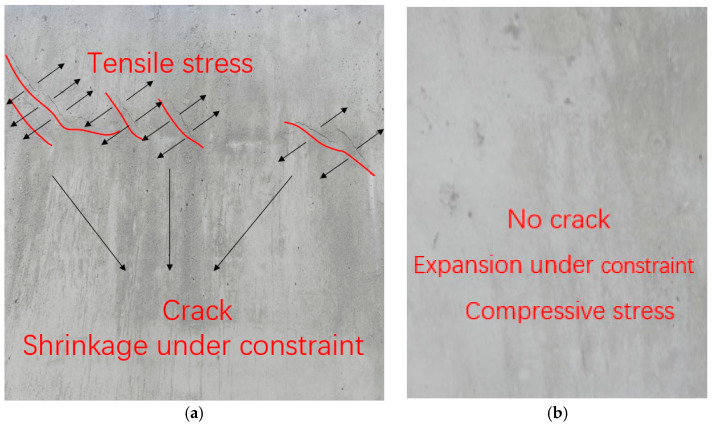
Distribution of cracks on the surface of reinforced concrete walls at 28 days. (**a**) CR, (**b**) CMM.

Figure 8 shows the distribution of cracks on the surface of the reinforced concrete walls at 28 days. The shrinkage of concrete was limited by steel bars and generated tensile stress. And cracks occurred when the tensile stress exceeded the tensile strength of concrete. At 28 days, many slender cracks (Figure 8a) were distributed on the surface of CR, which indicated that there was a large tensile stress due to shrinkage in the concrete. On the other hand, since the expansion of MEA compensated for the contraction, and the compressive stress was generated under the constraint conditions, this would not cause cracks in the wall. At this time, the wall surface was smooth, and no cracks were found (Figure 8b).

According to classical computational mechanics, when a wall is constrained by a foundation, cracks appear at the bottom of the wall, and cracks are perpendicular to the foundation. However, in many large-volume concrete structures, cracks are often randomly distributed and do not always appear near the foundation [25,26]. In our opinion, this is due to the fact that the walls are affected not only by their own temperature, but also by sunlight, which is also related to the construction method. Moreover, the shrinkage of the wall is not evenly distributed, and cracks often appear first where the quality of concrete pouring is poor. Therefore, we speculate that the concrete shrinkage limited by the foundation is an important cause of wall cracking, but this is not the only reason, and it is likely that the reinforcement will also affect the shrinkage.

### 3.3. Degree of Hydration of MEA

**Figure 9:**
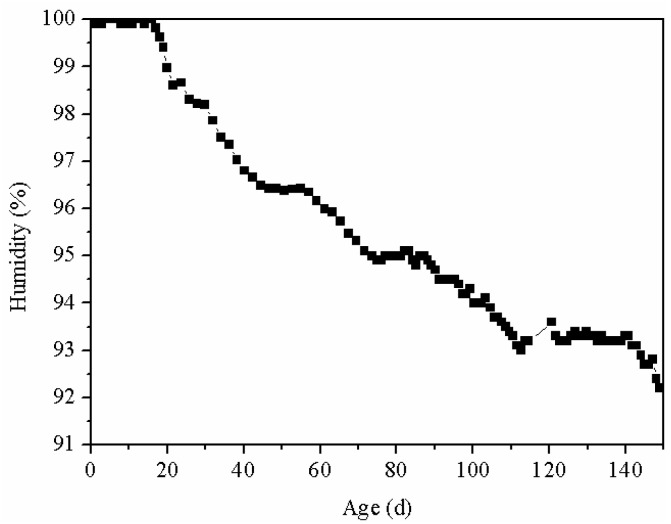
Moisture variation in the reinforced concrete wall.

The hydration of MEA depends on its own activity, maintenance temperature and humidity. As shown in Figure 9, owing to the large thickness of the wall, up to 0.8 m, the water inside the wall was hard to volatilize, which lead to the humidity inside the wall to remain at a high level. The relative humidity after 150 days still reached 92%, indicating that humidity was not the main factor affecting MEA hydration in this experiment. Therefore, this paper mainly discussed the effects of activity and temperature on MEA hydration.

**Figure 10:**
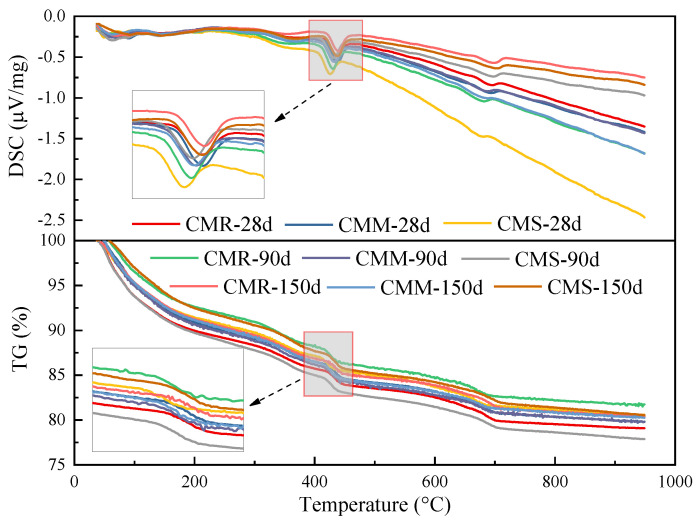
DSC/TG curves of MEA cement paste.

**Figure 11:**
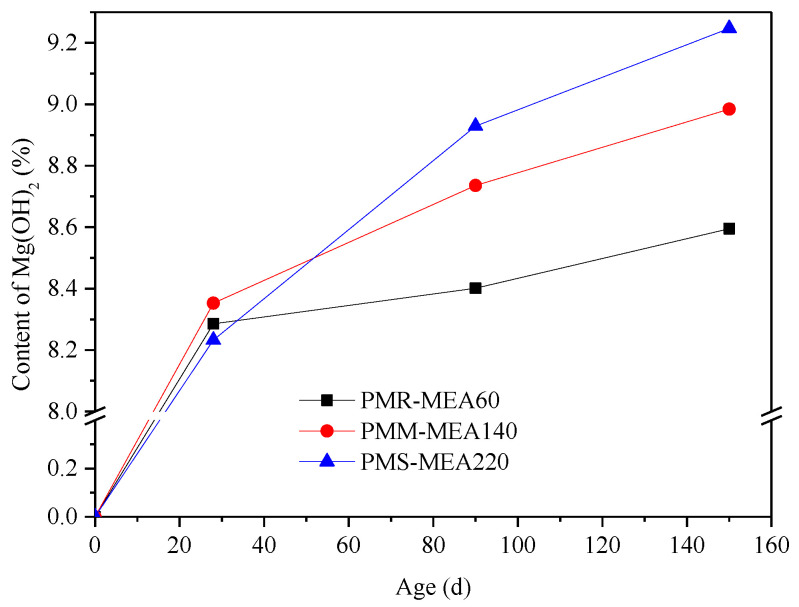
Content of magnesium hydroxide in the cement paste.

Figure 10 shows DSC/TG curves of the MEA cement paste buried in reinforced concrete walls at different curing age. According to the figure, the dehydration peak of magnesium hydroxide was 310~400 °C. Equation (2) was used to calculate the content of Mg(OH)_2_ in the cement paste, and the calculated results were shown in Figure 11. The hydration degree and Mg(OH)_2_ content of the MEA at different curing ages are showed in Table 8. As shown in Table 8, the hydration rate of the MEA at the early stage (0–27 days) was very fast, and the hydration degree of the three samples all exceeded 89%, specifically 96.4%, 93.0% and 89.0%, respectively. This was mainly owing to the fact that a large amount of heat was released during cement hydration at the early stage, and the high temperature accelerated the hydration rate of MEA, which was consistent with Li Hua’s research results. He found that curing temperature had a great influence on the MEA’s hydration and the hydration rate of the MEA at 80 °C was nearly 4.5 times of 20 °C at 28 days [27].

On the other hand, the activity of MEA also affected the hydration rate of MEA. At 28 days, compared with the content of Mg(OH)_2_ in cement paste with different activities of the MEA under the same content, Mg(OH)_2_ produced by MEA-S (PMS) with low activity was the least. However, at the later stage (160 days), PMS produced more Mg(OH)_2_ than the other two samples. As the expansion of the MEA was generated by MgO hydration to generate Mg(OH)_2_, it indicated that the expansion of the MEA with low activity was smaller in the early stage, but it produced greater expansion in the later stage, which was consistent with the laboratory test results of many other researchers [28,29].

### 3.4. Compressive Strength of Concrete

**Figure 12:**
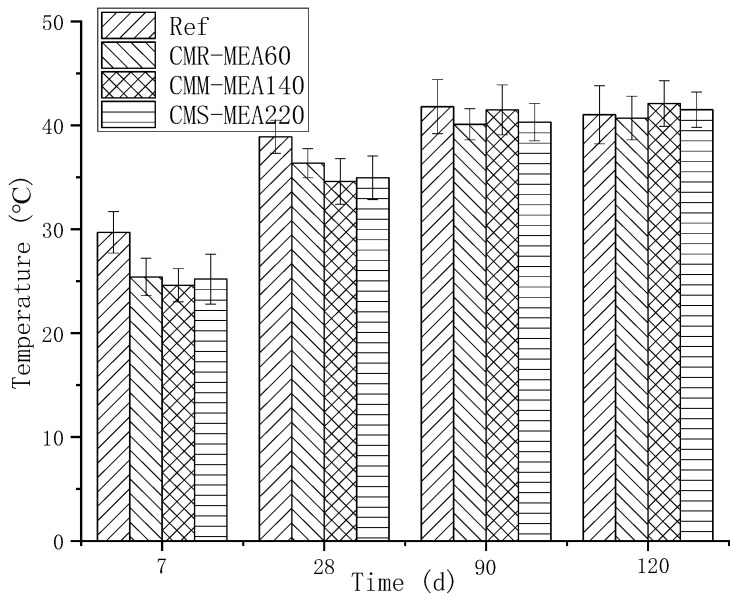
Compressive strength of pumped concrete.

Figure 12 shows the compressive strength variation of concrete. As the age of the concrete increased, the strength of the concrete with different mix proportions increased to different degrees. The growth rate of concrete in the early stage (0–28 days) was the highest, and the strength growth in the late stage gradually slowed down. For CR, the 28 days strength was increased by 31% compared to the 7 days strength, and the 90 days strength was only increased by 7% compared to the 28 days strength.

As some fly ash and mineral powder were replaced by the MEA, the amount of cementitious materials in concrete was reduced. As a result, in the early days (0–28 days), compressive strength of three MEA concretes (CMR, CMM and CMS) were lower than CR. However, with the continuous hydration expansion of MEA in the later stage, the hardened concrete restricted the outward expansion of MEA and made the MEA turn into an inward compression, which reduced the cavity of concrete and made the concrete more compact, thus improving the compressive strength of concrete.

### 3.5. Pore Structure of Concrete

**Figure 13:**
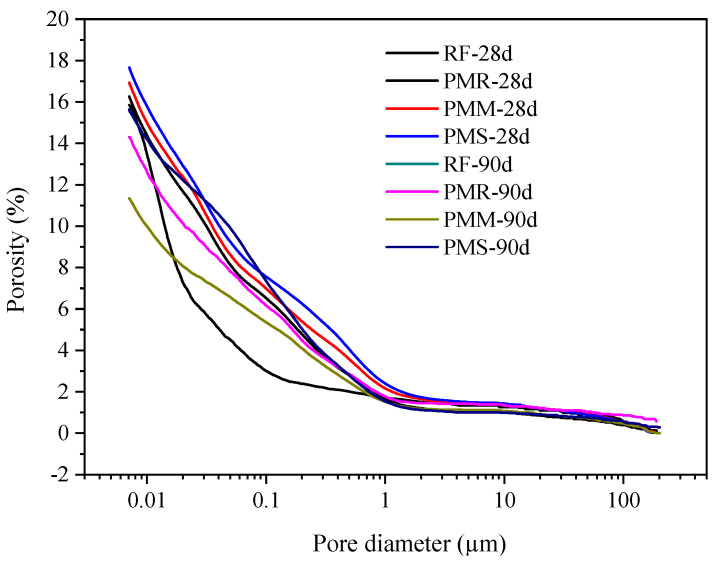
Porosity of cement paste of different ages.

Figure 13 and Table 9 illustrate the distribution of cement paste pores inside the concrete wall. The porosity of cement paste decreased with the growth of curing age. In the early stage (0–28 days), owing to the low strength of cement paste at this time, the outward expansion of the MEA was not effectively constrained. The MEA showed outward expansion, and the adjacent particles of cement paste were far away from each other. As a result, relative to PR, the porosity of the MEA cement was larger, which reduced the compressive strength of concrete in the early stage. In the later stage (90 days), owing to the expansion of the MEA being restrained by the hardened concrete, the MEA changed from outward expansion to inward compression deformation, which made the concrete denser. At 90 days, the porosity of the MEA140 cement paste decreased by 5.59%, while PR only decreased by 1.92%. Therefore, we believe that compared with plain concrete, the MEA can only compensate for shrinkage and cannot improve the strength of concrete. For reinforced concrete structures, due to the constraints of steel bars, self-stress is generated, which can also improve the compactness and strength of concrete [30,31].

## 4. Conclusions

(1) After 24 h of wall pouring, the temperature of the mass-reinforced concrete wall dropped sharply, which made the concrete produce a large shrinkage period and was prone to cracks. Therefore, effective measures must be taken to avoid wall cracking, ensure the safety of the wall and improve the durability of the wall.
(2) The shrinkage of reinforced concrete wall under the constraints of the foundation created tensile stress in the structure, resulting in cracks in the walls. Adding an MEA could effectively reduce the shrinkage of concrete, improve the volume stability of concrete, and reduce the generation of cracks.
(3) The effect of an MEA with different activities to compensate for the shrinkage of reinforced concrete walls is different. For MEA-R, because the activity is too high, its hydration rate is too fast, and many expansions occur at the plastic state of the concrete, which cannot effectively compensate for the shrinkage of concrete. For MEA-S, due to its low activity, the early hydration rate is so slow that it cannot compensate for the shrinkage, but it compensates well at the later stage due to the continuous hydration expansion of MEA. For MEA-M, the shrinkage of concrete is well compensated for the shrinkage at the early, middle and late stages due to its moderate activity.
(4) The early strength of the MEA concrete was lower than that of concrete without an MEA, and the early porosity is also higher. With the later expansion of the MEA, the porosity of the MEA decreased rapidly, and the strength also increased significantly, which indicates that the MEA will not reduce the strength while supplementing the shrinkage.
(5) Unlike plain concrete, an MEA can only compensate for shrinkage and cannot improve the strength of concrete. For reinforced concrete structures, due to the constraints of steel bars, self-stress is generated, which can also improve the compactness and strength of concrete. Therefore, MEA140 is safe and reliable for reinforced concrete walls of subway stations.

## Figures and Tables

**Table 1:** Chemical composition of cement.

ID	Chemical Composition/wt%									
	Loss	SiO_2_	Fe_2_O_3_	Al_2_O_3_	CaO	MgO	K_2_O	Na_2_O	SO_3_	Total
Cement	1.99	19.50	4.55	3.48	65.42	2.55	0.46	0.22	1.53	99.70

**Table 2:** Chemical composition of fly ash.

ID	Chemical Composition/wt%									
	Loss	SiO_2_	Fe_2_O_3_	Al_2_O_3_	CaO	MgO	K_2_O	Na_2_O	SO_3_	Total
Fly ash	4.52	41.84	8.31	36.71	4.40	1.92	0.68	0.40	0.52	99.30

**Table 3:** Chemical composition of mineral powder.

ID	Chemical Composition/wt%									
	Loss	SiO_2_	Fe_2_O_3_	Al_2_O_3_	CaO	MgO	K_2_O	Na_2_O	SO_3_	Total
Mineral powder	6.52	36.84	4.31	19.81	22.40	3.92	0.68	0.40	4.52	99.40

**Table 4:** Chemical composition of MEA.

ID	Activity Value/s	Chemical Composition/wt%						
		Loss	SiO_2_	Fe_2_O_3_	Al_2_O_3_	CaO	MgO	Total
MEA-R	60	5.17	4.26	0.98	1.34	3.87	83.73	99.35
MEA-M	140	4.49	4.45	0.42	0.73	3.19	85.44	98.72
MEA-S	220	3.16	4.28	0.45	0.78	3.01	87.81	99.49

**Table 5:** Mix proportions of C40 concrete/wt%.

ID	Cement	MEA	Fly Ash	Mineral Powder	Fine Aggregate	Coarse Aggregate		Water Reducer	Water
						5–16 mm	16–31.5 mm		
C-Ref (CR)	290	0	70	70	608	750	300	8.5	172
C-MEA-R (CMR)	290	34.4	52.8	52.8	608	750	300	8.5	172
C-MEA-M (CMM)	290	34.4	52.8	52.8	608	750	300	8.5	172
C-MEA-S (CMS)	290	34.4	52.8	52.8	608	750	300	8.5	172

**Table 6:** Mix proportions of cement paste embedded in reinforced concrete wall/wt%.

ID	Cement	MEA-R	MEA-M	MEA-S	Fly Ash	Mineral Powder	Water Reducer	Water
P-Ref (PR)	290	0	0	0	70	70	8.5	172
P-MEA-R (PMR)	290	34.4	0	0	52.8	52.8	8.5	172
P-MEA-M (PMM)	290	0	34.4	0	52.8	52.8	8.5	172
P-MEA-S (PMS)	290	0	0	34.4	52.8	52.8	8.5	172

**Table 7:** Volume deformation of reinforced concrete wall at different stages/με.

ID	Position 1			Position 2			Position 3		
	First Stage	Second Stage	Third Stage	First Stage	Second Stage	Third Stage	First Stage	Second Stage	Third Stage
CR	−138	−169	−235	−106	−132	−191	−50	−102	−149
CMR	−23	−32	−73	3	5	−42	−19	−52	−98
CMM	39	75	40	36	73	46	19	29	−8
CMS	−25	23	−17	−14	10	−27	−7	14	−25

**Table 8:** Variation of hydration degree of cement with age in samples.

ID	Content of Mg(OH)_2_/%			Hydration Degree/%		
	28 d	90 d	150 d	0~28 d	28~90 d	90~150 d
PMR	8.29	8.40	8.59	96.4	1.3	2.3
PMM	8.35	8.74	8.98	93.0	4.3	2.7
PMS	8.23	8.93	9.25	89.0	7.5	3.5

**Table 9:** Distribution of cement paste pores in concrete walls (%).

No.	Porosity	<10 nm	10~50 nm	50~500 nm	>500 nm
PR-28d	15.85	2.39	8.98	2.48	2.00
PMR-28d	16.26	1.87	6.26	5.39	2.74
PMM-28d	16.93	1.93	6.38	5.13	3.49
PMS-28d	17.66	1.88	6.56	5.32	3.90
PR-90d	13.93	1.31	4.8	4.97	2.85
PMR-90d	14.31	1.69	4.80	4.97	2.85
PMM-90d	11.34	1.35	3.41	4.14	2.44
PMS-90d	15.63	1.43	4.29	7.14	2.77

## Data Availability

All data generated or analyzed in this research were included in this published article. Additionally, readers can access all data used to support conclusions of the current study from the corresponding author upon request.
